# A 30,000-km journey by *Apus apus pekinensis* tracks arid lands between northern China and south-western Africa

**DOI:** 10.1186/s40462-022-00329-2

**Published:** 2022-06-29

**Authors:** Yanyan Zhao, Xinru Zhao, Lan Wu, Tong Mu, Fang Yu, Lyndon Kearsley, Xuan Liang, Jianping Fu, Xiaoru Hou, Peng Peng, Xiaoyang Li, Tao Zhang, Su Yan, Dick Newell, Chris M. Hewson, Terry Townshend, Susanne Åkesson, Yang Liu

**Affiliations:** 1 State Key Laboratory of Biocontrol, School of Ecology, Sun Yat-Sen University, Guangzhou, 510275 Guangdong China; 2 Institute of Eco-Environmental Research, Guangxi Academy of Sciences, Nanning, 530007 Guangxi China; 3 Beijing Normal University, Beijing, 100875 China; 4 China Bird Watching Society, Beijing, 100097 China; 5 Department of Ecology and Evolutionary Biology, Princeton University, Princeton, NJ 08544 USA; 6 Belora Vzw, Kloetstraat 48, B-9120 Melsele, Belgium; 7 Administrate Office of the Summer Palace, Beijing, 100080 China; 8 Action for Swifts, Old Beach Farm, 91 Green End, Landbeach, Cambridge, CB25 9FD UK; 9 British Trust for Ornithology, The Nunnery, Thetford, Norfolk, IP24 2PU UK; 10 Department of Biology, Center for Animal Movement Research, Lund University, Ecology Building, 223 62 Lund, Sweden

**Keywords:** Common Swift, Migration, Light-level geolocator, Population divergence, East Asia

## Abstract

**Background:**

As a widely distributed and aerial migratory bird, the Common Swift (*Apus apus*) flies over a wide geographic range in Eurasia and Africa during migration. Although some studies have revealed the migration routes and phenology of European populations, *A. a. apus* (from hereon the nominate *apus*), the route used by its East Asian counterpart *A. a. pekinensis* (from hereon *pekinensis*) remained a mystery.

**Methods:**

Using light level geolocators, we studied the migration of adult *pekinensis* breeding in Beijing from 2014 to 2018, and analysed full annual tracks obtained from 25 individuals. In addition, we used the mean monthly precipitation to assess the seasonal variations in humidity for the distribution ranges of the nominate *apus* and *pekinensis*. This environmental variable is considered to be critically relevant to their migratory phenology and food resource abundance.

**Results:**

Our results show that the swifts perform a round-trip journey of ca 30,000 km each year, representing a detour of 26% in autumn and 15% in spring compared to the shortest route between the breeding site in Beijing and wintering areas in semi-arid south-western Africa. Compared to the nominate *apus*, *pekinensis* experiences drier conditions for longer periods of time. Remarkably, individuals from our study population tracked arid habitat along the entire migration corridor leading from a breeding site in Beijing to at least central Africa. In Africa, they explored more arid habitats during non-breeding than the nominate *apus.*

**Conclusions:**

The migration route followed by *pekinensis* breeding in Beijing might suggest an adaptation to semi-arid habitat and dry climatic zones during non-breeding periods, and provides a piece of correlative evidence indicating the historical range expansion of the subspecies. This study highlights that the Common Swift may prove invaluable as a model species for studies of migration route formation and population divergence.

**Supplementary Information:**

The online version contains supplementary material available at 10.1186/s40462-022-00329-2.

## Background

Bird migration, connecting remote places, has captured the attention of humans for thousands of years, yet systematic scientific research only started about a hundred years ago [1]. It is generally believed that evolution of migration routes is driven and constrained by both external (ecological and biogeographic factors) and inherited factors (genetic components) [2]. This may lead to population divergence and speciation in long-distance migratory organisms [3], and some evidence of this has been found in several species, such as a long-distance migratory passerine, the Barn Swallow (*Hirundo rustica*) [4, 5]. Glaciation periods caused by historical climate change may have led to fluctuations in population distributions (e.g. expansion) [6], thus playing an important role in shaping current migration routes and strategies [7, 8], and further have an impact on population divergence [9]. Modern climatic conditions (e.g. temperature, rainfall and wind) may also affect the movement and survival of long-distance migrants [10, 11], of these, precipitation can affect the migration performance and survival rate of migrants, especially for aerial insectivorous birds, by changing the abundance and distribution of food resources [12, 13].

As a typical long-distance migratory and aerial insectivore, the Common Swift (*Apus apus*) is widely distributed in the Palearctic and often nests in cavities in buildings [14]. It has two subspecies: the nominate *apus* which breeds more northerly, from Europe and north-western Africa through to northern Asia, and the eastern subspecies *pekinensis* (also known as ‘Beijing Swift’) breeds from western/central Asia to eastern Asia [14, 15]. The easternmost breeding range of *pekinensis* overlaps with two other migratory swifts, i.e. the Pacific Swift (*Apus pacificus*), and the White-throated Needletail (*Hirundapus caudacutus*), whose migratory paths have been recently elucidated by light-level geolocators [16, 17], virtually following the East Asian-Australasian Flyway (EAAF) [18].

The Common Swift is an emerging model organism for the study of bird migration. In particular, several studies using different types of data loggers in European populations have revealed their migration routes, migration strategies and their inter-annual variation, unusual chain-migration patterns and possible drivers [19–24]. In contrast, the knowledge of migration behaviour and route choices of the eastern subspecies *pekinensis* is poorly understood. Documented specimens and field records have been collected from arid south-west Africa to southern Angola, Namibia and Botswana, and a small extent as far north as Zaire, Uganda, Sudan and United Arab Emirates [25, 26]. This evidence suggests a southern non-breeding distribution in Africa, also raising the possibility that *pekinensis* could follow an Asian-African flyway [27]. Yet the information hardly provides details on the migratory pathway en route to their breeding grounds in East Asia.

It is believed that the nominate *apus* and *pekinensis* can be reliably separated by some morphological features [27]. The breeding ranges of the two subspecies are parapatric, and a contact zone is traditionally considered to stretch from east of Lake Baikal to Iran through central Kazakhstan [28]. For long-distance migrants, seasonal migrations pose big challenges not only due to the high energy cost of flight, but also due to phenotypic and physiological adaptations to likely changeable environments between breeding and non-breeding habitats. Divergence in migratory routes, phenology and strategies can act as a prezygotic isolation mechanism to population differentiation between closely related migratory taxa [3, 29]. This may well be the case for the Common Swift. A recent study revealed that the northern population of nominate *apus* winters in west and central sub-Saharan Africa, whereas the southern European *apus* swifts spend winters in contiguous regions of central and southeastern Africa [22]. It suggests that, even within the European breeding populations of the nominate *apus*, there are substantial spatiotemporal separation in wintering grounds, as well as migratory phenology [22]. Therefore, uncovering the approximate wintering ground and detailed migratory route of *pekinensis* can help us understand the migration patterns of the East Asian population of the Common Swift, which is the prerequisite to allow comparisons of migration phenotypes and to make inferences of divergence between the nominate *apus* and *pekinensis*.

In this study, we characterized the migration route and phenology of *pekinensis* using light-level geolocators that were deployed during five breeding seasons at the Summer Palace, Beijing, China (a UNESCO World Heritage site). The study population is located almost at the easternmost part of the geographical range, and we expect that they might travel the longest migration distance between its potential breeding and nonbreeding areas as compared to the nominate *apus*. In turn, we expected that *pekinensis* exhibit a different migration strategy in terms of route and penology in comparison to the nominate subspecies. Because long-distance bird migrants commonly track seasonal resources between breeding and wintering grounds [30], we further hypothesized that it is highly likely *pekinensis* and *apus* experience very different environmental conditions throughout their migration. To this end, we tested the differences in precipitation along the migratory flyways between *pekinensis* and the nominate *apus*, since the amount of rainfall has been regarded as one key climatic factor that negatively correlate with swift’s survival [31, 32]. Together, this study complements our knowledge of the migratory strategies of an aerial long-distance migrant, and towards understanding population divergence in long-distance migratory birds.

## Methods

### Field procedure

We carried out fieldwork at the Kuoru Pavilion (116.2726°E, 39. 9891°N) situated in the Summer Palace in Beijing, China. Once per breeding season from 2014 to 2018, we captured breeding swifts using mist nets arranged around the pavilion between 03:45 and 06:00 on a single day around May 22th. Upon first capture, randomly selected individuals were fitted with light-level geolocators (0.67 g, Intigeo-W65C1, Migrate Technology Ltd.) using a full body harness made of soft braided flat nylon string (1 mm wide, average harness weight 0.09 g) around the neck and both wings [19], the total mass of the geolocators and harness is 2.04 ± 0.09% of the body mass of the birds. Using blood lancet and capillary, an extraction of 50 μl of blood was also collected from each individual’s brachial vein, and stored in 99% ethanol and a -80℃ freezer for the following molecular sex determination.

In this study, a total of 66 swifts were outfitted with geolocators of which 22 were retrieved the following year. We also banded 250 birds without attaching geolocators and recaptured 131 after one year. The average recovery rate with geolocator across four years was 30.0% (range 20.0–41.9%), which was lower than that in Sweden (ca 50%), but approximately close to those from other colonies (ca 30%) [33], and lower than the average recapture rate without geolocator in this study (53.4%, range 36.2–65.5%). Although there is evidence that light-level geolocators have a negative impact on the survival rate of swifts during migration [33], we assume that the very limited banding effort (i.e. we were allowed one 3-h capture period on a single day per year at this site), may be the most important reason for the low recovery rate in this study.

Finally, we successfully recaptured a total of 25 geolocators by 2018 including five birds with two-years of tracking data (three retrieved in the third year and two retrieved twice in the following two years). To allow consistent comparisons [24], we retained only the first year of data for the five individuals. Finally, 25 tracks covering both complete autumn and spring migrations were included in the analysis. In addition, the sex of fifteen males and six females were identified by a standard molecular sexing method [34], and four individuals were not sexed because no blood samples were taken at capture (Additional file 1: Table S1).

### Data analysis

#### Position calculation

The light-level data were analysed using *GeoLight* 2.0.0 [35], an easy-to-adjust, intuitive and easy-to interpret package in *R* 4.0.2 [36]. More importantly, it allows comparisons of results generated with other packages in similar principals [19, 21, 37]. We set the threshold to 2 for log-transformed light-level data to identify the twilight events (dawn-sunrise and sunset-dusk) minimizing the latitude variation around the equinox [19, 20, 22]. In the pre-analysis, we found that the swifts stayed in the breeding site for a very short and variable period before and after breeding. Thus we used the “Hill-Ekstrom” procedure [38] to determine the single sun elevation angle for position estimating through the whole migration cycle for each individual [19, 20], which ranged from −4.8 to −6.6° (Additional file 2: Table S6). We excluded latitude estimates from two weeks before and after the spring and autumn equinoxes, as accuracy for latitude determination during this time is low. The movement state (i.e. stationary, directed flight) during these periods was estimated by changes in longitude and positions before and after [20, 37]. The final position data were imported into *QGIS* 3.14 for further analysis.

#### Stopover sites

Given the error margin of the light-level geolocators [39, 40], especially for the fast-flying species such as swifts, we used the *changeLight* function (quantile = 0.95, days = 3) [35] to determine the stopovers (local ranging). To avoid the inaccuracy of some positions caused by the occasional abnormal twilight events, we carried out manual correction for outliers with *QGIS*. Since the reported travel speed of Common Swift was above 250 km/day, and the stopovers below 2 days were considered indistinguishable from slow movement due to the inaccurate positioning [19, 21, 37], locations changing less than 500 km in at least 3 days were grouped as ‘stopover sites’ in our study. Then we selected the first position after the swifts reaching a stopover site to define the arrival date, and the last point before they leaving to define the departure date, and calculated the stopover time from that.

#### Autumn migration, winter quarters and spring migration

During the wintering period, the swifts were constantly moving around covering short distances per day, forming restricted “residence areas” with distinct arrival and departure paths, which were defined as wintering areas. The *pekinensis* swifts might move northeast in the later part of winter, often causing the central position of wintering area and median time of wintering period to be out of sync. Therefore, we chose the first position after the swifts entering the wintering area to define the end of autumn migration, and the last position before leaving to define the start of spring migration. The dates of arrival and departure from the wintering area were simultaneously determined in this step.

Fast east–west movements change the perceived day and night lengths the swifts experienced, interfering with accurate positioning, particularly latitude, which was more obvious during spring migration, so the end of the spring migration was determined by the first point less than 500 km away from Beijing, or at 116°E (longitude of Beijing).

#### Total migration distance

In order to reduce the effects of uncertainty associated with location estimates using geolocator data, we used the distances connecting 3-day average positions to calculate the total migration distance [19]. We also calculated the great circle route distance between the start and end points of each migration as the direct distance. Positions of stopover sites were replaced by the average locations during the stopover periods. The average migration speed was calculated including the stopover periods, while the travel speed was calculated excluding them. Most of the swifts in our study showed relatively long stays in or near the Congo Basin, which was considered by several studies to be part of the overwintering period [21], as the tracking of migratory birds for seasonal resources [30]. In order to describe the whole migration process, we selected the farthest and longest overwintering sites as endpoints to calculate the parameters of migration phenology.

We compared the sexual and seasonal variations of migration parameters using t-tests. In addition, we compared the migration phenology of two groups of individuals from different subspecies, using a detailed set of published data from Sweden (N = 25) [19, 37],representing northernmost populations of *apus*. In addition, we also compared our data with some general information on movement patterns from several European populations of *apus* in published studies which also used farthest sites as migration endpoints [22].

#### Precipitation in the distribution regions

We assessed the condition of aridity in both reported and randomly selected positions throughout the distribution ranges using monthly precipitation from 2014 to 2018. Since the populations of the nominate *apus* were known to have different migration patterns in the northern and southern European populations, we compared them with *pekinensis* from Beijing respectively. Firstly, we extracted the precipitation data of four breeding sites of *pekinensis* with phenological records [41–43], including Beijing and three sites less than 500 km from Beijing, and that of 24 breeding sites of nominate *apus* in central and western Europe (8 northern, and 16 southern) [22] (Additional file 1: Table S5). In addition, given the limited number of sites with reported phenology information, we randomly took 100 points respectively in the breeding ranges of the two subspecies with a distance greater than 2 degrees between each two adjacent points to assess the precipitation in their entire breeding areas [15, 44] (Additional file 3: Fig. S1A; Additional file 4: Table S7). According to our study and the literature records [19, 22], we selected two time periods, April to July for *pekinensis* and May to August for nominate *apus*, to compare the mean monthly precipitation experienced by the two subspecies in breeding areas. Secondly, since the positions of Common Swifts are not fixed during the wintering period, we used the precipitation of the corresponding month at each position [22] to calculate the average value (Additional file 5: Table S8). Since the location information available for populations of nominate *apus* is incomplete during the wintering period, we only take the precipitation experienced by the three populations in November and December for comparison. Similarly, we used the random point method to measure the precipitation in the whole wintering areas, and determined the wintering periods and ranges of the two subspecies: November to February for *pekinensis*, October to April for northern and November to February for southern populations of nominate *apus* [19, 21, 22]. We randomly and evenly took 50 points respectively in the wintering ranges of three geographical populations (Additional file 3: Fig. S1B; Additional file 6: Table S9). Finally, we compared the mean monthly precipitation during the breeding and wintering periods at both “reported sites” and “random sites” between the two geographical populations of nominate *apus* and *pekinensis* as well as the annual precipitation at “random sites”, and compared the mean monthly precipitation during the wintering period of the three populations between the “reported sites” and “random sites”. The historical precipitation was obtained from WorldClim 2.1 [45] (https://www.worldclim.org), with a resolution of 2.5 min.

Key parameters of migration and precipitation were extracted, and maps were made in *QGIS* 3.14. All statistics were carried out in *R* 4.0.2 [36].

## Results

### Migration route

**Fig. 1:**
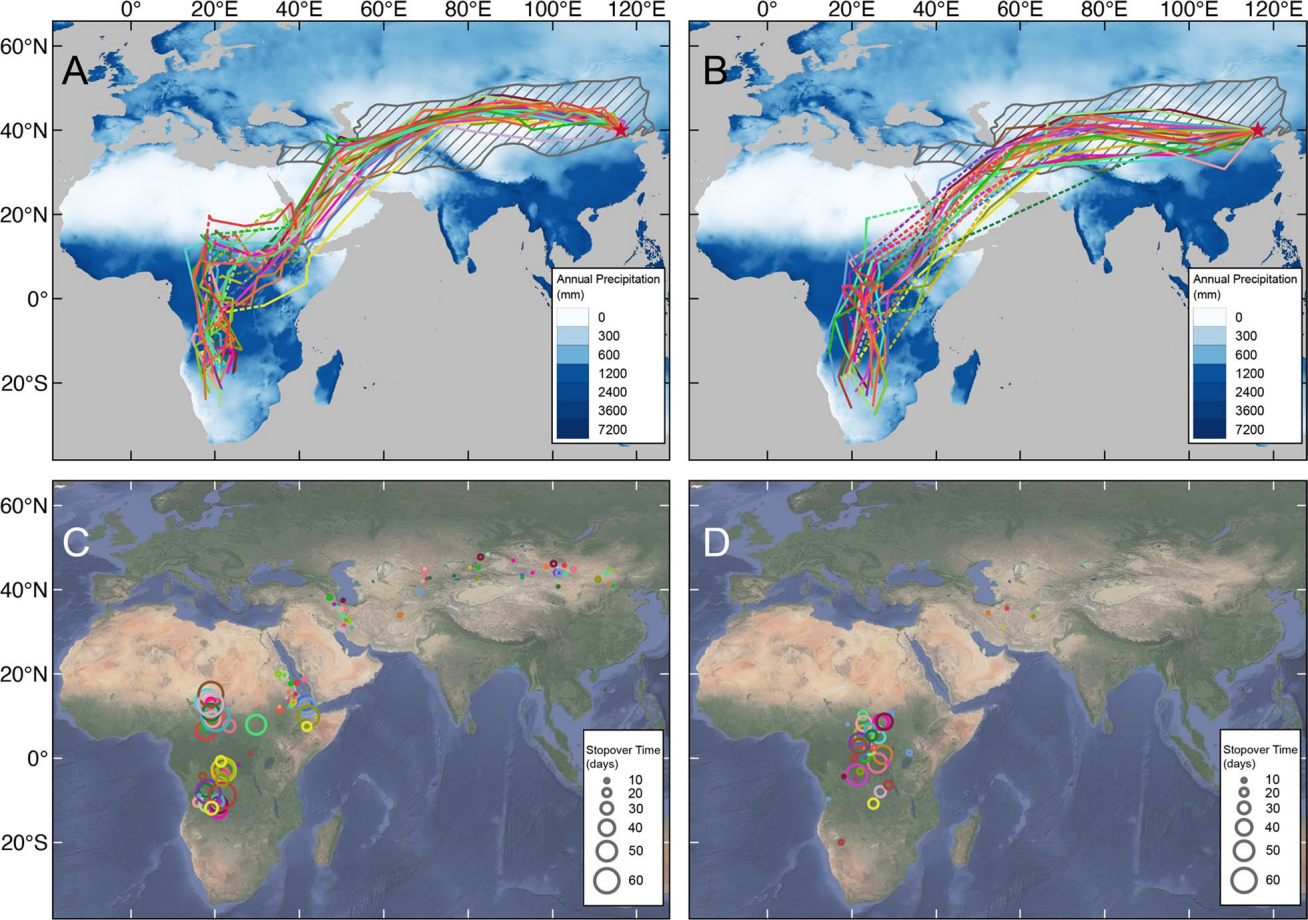
Maps showing the migration routes and stopover sites of *A. a. pekinensis*. N = 25 (for details see Additional file 7: Fig. S2). In **A** (autumn) and **B** (spring): the gray hatching shows the breeding distribution of *pekinensis* [15, 44], the dotted lines indicate the lack of data in the two weeks before/after the equinoxes, and the base map shows the global annual precipitation distribution. The circles in **C** (autumn) and **D** (spring) represent the stopover sites. Different colour symbols represent different individuals. The pentagram represents the *pekinensis* breeding site and fieldwork location in Beijing.

Around July 17th (range: July 3rd–25th) (Table 1), the tagged swifts left Beijing after breeding and initially departed towards the northwest into Mongolia, and thereafter moved westwards. After this initial migration period they re-entered China, passing through northern Xinjiang, and entered central Asia through the Junggar Basin between the Altai and Tienshan Mountains (Fig. 1; Additional file 7: Fig. S2).

**Table 1:** Key phenological parameters of migration of *A. a. pekinensis* breeding in Beijing (N = 25)

	Mean ± SD	Range
*Autumn migration*		
Departure from Beijing	Jul 17th ± 6	Jul 3rd–Jul 25th
Travel time (days)	40 ± 14	22–69
No. stopover sites	4 ± 1	2–6
Stopover time (days)	71 ± 15	39–104
Total duration (days)	111 ± 13	89–134
Migration distance (km)	14,733 ± 775	13,432–16,096
Direct distance (km)	11,738 ± 302	11,238–12,360
Detour (%)	25.56 ± 6.71	15.35–37.31
Travel speed (km/day)	423 ± 171	198–981
Migration speed (km/day)	134 ± 17	109–170
Arrival at wintering area	Nov 5th ± 11	Oct 16th–Nov 24th
Duration of wintering period (days)	100 ± 16	67–139
*Spring migration*		
Departure from wintering area	Feb 13th ± 12	Jan 16th–Mar 6th
Travel time (days)	29 ± 11	14–56
No. stopover sites	1 ± 1	1–4
Stopover time (days)	36 ± 12	3–58
Total duration (days)	64 ± 11	38–90
Migration distance (km)	13,572 ± 999	12,411–16,321
Direct distance (km)	11,817 ± 281	11,202–12,328
Detour (%)	14.85 ± 8.00	3.32–36.26
Travel speed (km/day)	528 ± 171	229–910
Migration speed (km/day)	217 ± 34	156–327
Arrival at Beijing	Apr 18th ± 9	Apr 7th–May 14th
Duration in Beijing (days)	90 ± 10	71–109

**Fig. 2:**
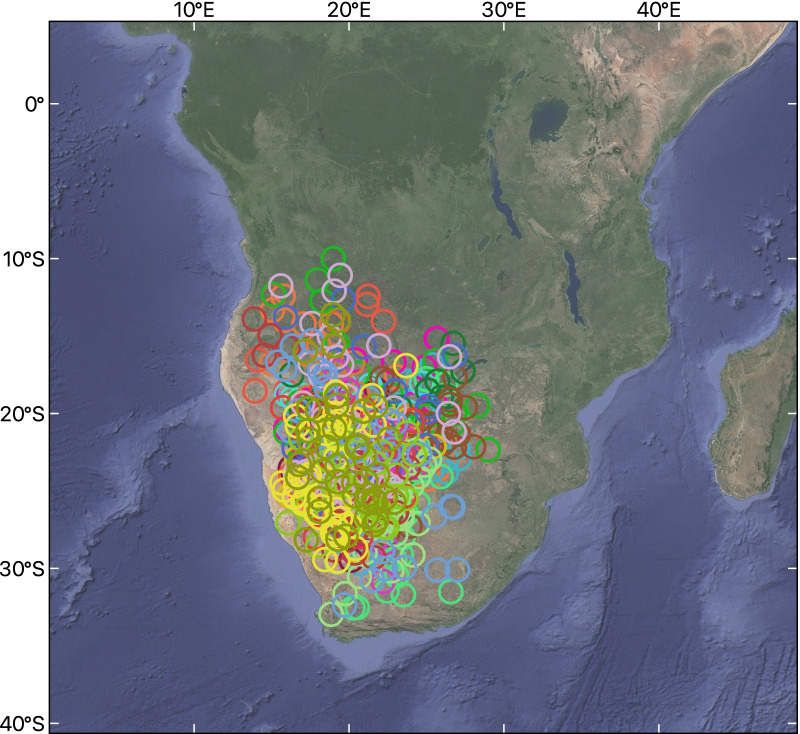
Map showing the locations of *A. a. pekinensis *in wintering area. The circles represent the 3-day average positions of different individuals during the wintering period. Different colour symbols represent different individuals

From central Asia, the swifts migrated to north-eastern Africa with three main stopovers explored in this region. They crossed the Red Sea around August 16th (± 11 days, range: July 27th–September 9th). Thereafter they moved to central Africa, reaching the approximate longitude of the eastern Congo Basin in early September, where they remained for around 39 ± 16 days (mean ± SD) before slowly moving south. Due to the Autumnal Equinox, we were not able determine how the locations of most individuals during this period relate to the Congo Basin. The swifts reached the Southern African Plateau (on average 1000 m asl) around November 5th. There, the swifts stayed for 100 ± 16 days, roaming the area before moving northeast around February 13th (Table 1, Fig. 2).

Soon after the onset of spring migration, the *pekinensis* swifts reached the eastern Congo Basin in mid-February, and stayed there for about one month (25 ± 20 days). Then they crossed the Red Sea, leaving Africa in early April, and flew back to the breeding area almost nonstop, arriving in Beijing on average at April 18th (± 9 days, range: April 7th–May 14th, with only one individual arriving in May) (Table 1).

The migration distance in autumn was 14,733 ± 775 km, which was significantly longer than in spring (13.572 ± 999 km) (t = 4.59, df = 24, *p* < 0.001) (Table 1). The average detour during autumn migration was 25.56 ± 6.71%, which was also bigger than that during spring migration (14.85 ± 8.00%) (t = 5.23, df = 24, *p* < 0.001).

### Migration phenology

The difference in migration duration, speed and stopovers were significant between autumn and spring. In autumn, the average duration of migration was 111 ± 13 days, significantly longer than 64 ± 11 days in spring (t = 11.85, df = 24, *p* < 0.001). Therefore, the average total migration speed in autumn, 134 ± 17 km/day, was significantly slower than 217 ± 34 km/day in spring (t = −9.52, df = 24, *p* < 0.001). Excluding the stopover periods, the average travel speed in autumn was 423 ± 171 km/day, which was slightly slower than 528 ± 171 km/day in spring (t = −1.88, df = 24, *p* = 0.07) (Tables 1 and 2).

**Table 2:** Results of comparative analysis of characteristic parameters between autumn and spring using paired t-test

	t	df	*p *value
*All *(*N = 25*)			
Migration distance	4.59	24	**1.18e−04**
Detour	5.23	24	**2.31e−05**
Travel speed	− 1.88	24	0.07
Migration speed	− 9.52	24	**1.27e−09**
Total duration	11.851	24	**1.62e−11**
Stopover time	7.66	24	**6.75e−08**
Travel time	2.66	24	**0.01**
*Female *(*N = 6*)			
Migration distance	0.42	5	0.69
Detour	0.55	5	0.61
Travel speed	− 2.44	5	0.06
Migration speed	− 6.02	5	**0.002**
Total duration	4.84	5	**0.005**
Stopover time	1.94	5	0.11
Travel time	2.40	5	0.06
*Male *(*N = 15*)			
Migration distance	4.74	14	**3.18e−04**
Detour	5.91	14	**3.78e−05**
Travel speed	− 1.41	14	0.18
Migration speed	− 8.15	14	**1.11e−06**
Total duration	9.40	14	**1.99e−07**
Stopover time	8.08	14	**1.23e−06**
Travel time	1.62	14	0.13

Significant effects are marked with bold

We found that the swifts on average used four stopover sites in autumn and only one site in spring, most of them in the central part of Africa, possibly in or near the Congo Basin. The locations of the other stopover sites were clustered, of which the southwest coast of the Red Sea and the south coast of the Caspian Sea were two areas with highly used stopover areas with several individuals (Fig. 1C and D). The average total stopover time in autumn was 71 ± 15 days, significantly longer than 36 ± 12 days in spring (t = 7.66, df = 24, *p* < 0.001) (Tables 1 and 2). Moreover, our study revealed no difference in timing, distance, duration, or speed of migration between sexes (Additional file 1: Table S2 and S3).

### Temporal and spatial distribution of precipitation

**Fig. 3:**
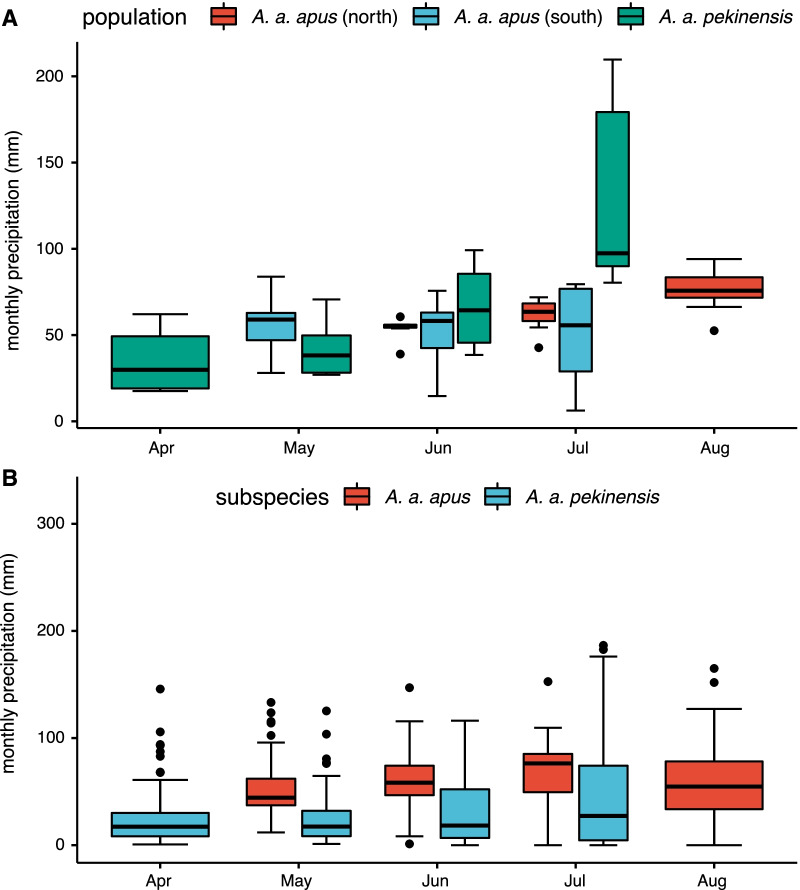
Distribution of monthly precipitation during breeding season of the two subspecies. **A**: reported sites; **B**: random sites

**Fig. 4:**
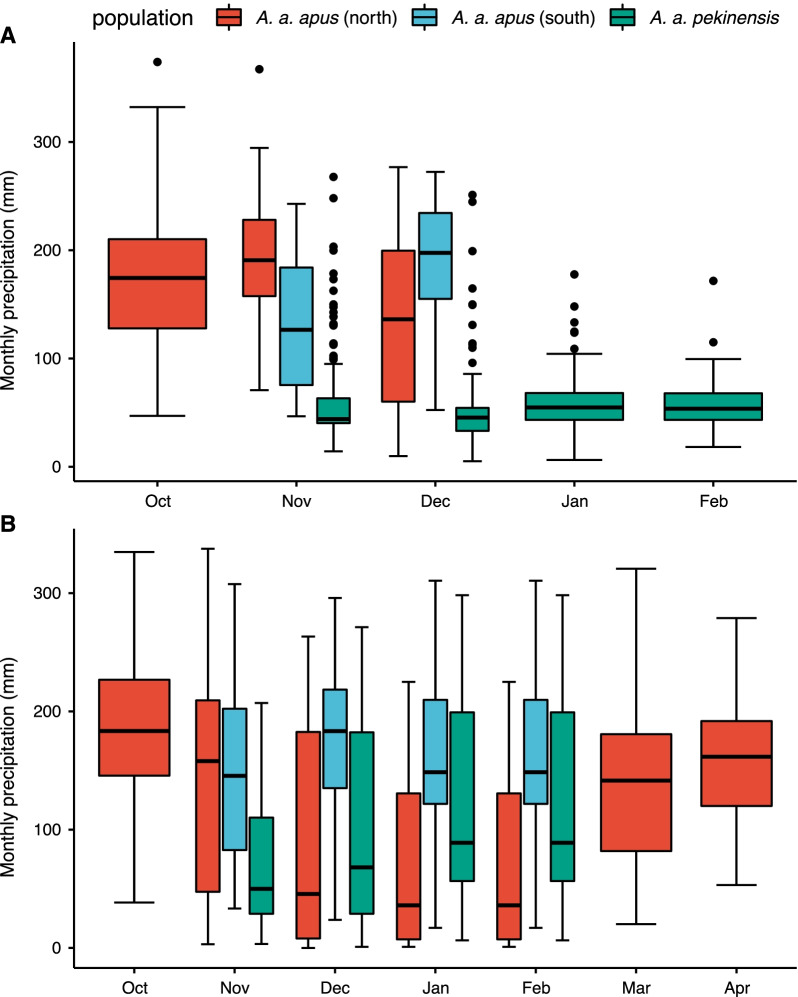
Distribution of monthly precipitation during wintering season of the two subspecies. **A**: reported sites; **B**: random sites

At breeding sites, where information about breeding and migration has been reported [22, 41–43], we did not find a significant difference in rainfall between groups (*pekinensis* vs. northern nominate *apus*: t = −0.43, df = 26.25, *p* = 0.67, and *pekinensis* vs. southern nominate *apus*: t = −1.45 df = 26.61 *p* = 0.16). Based on random sampling throughout the whole breeding areas, *pekinensis* experienced significantly lower rainfall (t = 11.40, df = 763.37, *p* < 0.001), as compared to ranges explored by nominate *apus* (Fig. 3). During the wintering period, *pekinensis* remained in significantly drier areas than nominate *apus*, shown both for the restricted sample of reported sites (*pekinensis* vs. northern nominate *apus*: t = 12.30, df = 86.30, *p* < 0.001; *pekinensis* vs. southern nominate *apus*: t = 13.97, df = 85.04, *p* < 0.001) and when the complete wintering ranges were considered (*pekinensis* vs. northern nominate *apus*: t = 2.27, df = 432.82, *p* = 0.02; *pekinensis* vs. southern nominate *apus*: t = 7.62, df = 390.53, *p* < 0.001) (Fig. 4).

The subspecies *pekinensis* tracked from Beijing spend 46.1% of their non-breeding period (123 ± 17 days, range 83–168 days) in areas with less than 600 mm of annual precipitation. This figure is much higher than that in the nominate *apus* from Sweden, which spent only 25 days mainly in the Sahara Desert (10.5% of non-breeding period) [19, 20]. In fact, the average annual precipitation for the distribution of *pekinensis* is also significantly lower than that for the nominate *apus* throughout its breeding range (284.01 ± 231.67 mm vs. 629.34 ± 292.66 mm; t = 9.25, df = 188.09, *p* < 0.001) and wintering area (*pekinensis*: 617.79 ± 421.54 mm; northern nominate *apus*: 1608.27 ± 396.29 mm; southern nominate *apus*: 1262.69 ± 378.70 mm; *pekinensis* vs. northern nominate *apus*: t = 12.05, df = 96.84, *p* < 0.001; *pekinensis* vs. southern nominate *apus*: t = 8.05, df = 96.90, *p* < 0.001), as calculated from random positions. When comparing the mean monthly precipitation between "reported sites" and "random sites" during the wintering period, we found that the precipitation experienced by these known individuals was different from that of the whole wintering area, with higher precipitation for northern nominate *apus* (t = 5.76, df = 243.39, *p* < 0.001), and lower for *pekinensis* (t = −8.46, df = 213.27, *p* < 0.001), but similar for southern nominate *apus* (t = -0.03, df = 147.03, *p* = 0.98).

## Discussion

### Differences in migration characteristics for *pekinensis* and nominate *apus*

Compared to the northern population of nominate *apus* breeding in Sweden for which migration routes and phenology have been well described [19, 22, 37], *pekinensis* from Beijing had a significantly longer breeding period, stopover time, migration distance and duration, shorter wintering period and lower movement speed, except not significantly for travel speed in spring (Additional file 1: Table S4). And compared to southern populations of nominate *apus* from several different sites, the migration distance of *pekinensis* in our study was nearly twice that of theirs [22]. Meanwhile, *pekinensis* spent 64% and 54% of their total migration duration at stopover sites in autumn and spring, respectively, which were both significantly higher than nominate *apus* from Sweden. As food resource and climate conditions at stopover sites are important for flight performance and survival during bird migration [46, 47], we assume it is likely that *pekinensis* spend more time accumulating fat reserves or ranging locally during migration in order to manage a longer migration journey [48]. As for the long suspected stopover in central Africa of *pekinensis*, more accurate tagging methods, such as GPS-tracking, are needed to pinpoint the location of the swifts and thus to understand their spatial use and ecology in the Congo Basin and surrounding plateaus.

The spring migration duration of *pekinensis* was much shorter than autumn with both faster migration and travel speeds, as found in nominate *apus* and many other migratory birds [19, 49]. The difference in migration speed between spring and autumn is usually explained as selection for early arrival due to intraspecific competition for mates and resources during the breeding season [50], which could be mainly mediated by seasonal variations in stopover duration [50, 51].

### Migration and subspecies divergence

Divergence in migratory routes in closely related populations/subspecies has been considered to be associated with population divergence [3, 52].The most important difference revealed is that the migratory range of *pekinensis* covers to a large extent semi-arid habitats in Continental Asia (Fig. 1). In this study, the wintering area of *pekinensis* and the nominate *apus* from central Europe partially overlapped in the Katanga Plateau [22]. But in general, *pekinensis* overwinters in areas with less rainfall. Even so, *pekinensis* still followed drier habitat while nominate *apus* followed wetter habitat in areas with more rainfall, which may indicate that *pekinensis* might have adapted to different climatic zones or have different patterns of food resource utilization during non-breeding period [53]. Some small parts of the breeding range of *pekinensis* have variable seasonal precipitation. For instance, the narrow areas in the far east near the ocean and the southwestern foothill of the Himalayas, have relatively higher rainfall from July to August. However, *pekinensis* here usually begin their autumn migration by this time, suggesting that this subspecies may have a relatively strong attachment to arid habitats, especially during non-breeding periods.

Taking a direct route from the wintering grounds meant that *pekinensis* would have to pass over the Arabian Sea and the Tienshan-Himalaya mountains. To avoid the two physical barriers, i.e. ocean and mountains [54–56], there is another similar-length potential route passing around the Arabian Sea and turning northeast below the Himalayas, which is used by other bird species that have similar breeding and wintering areas to *pekinensis* in eastern Asia, such as *Cuculus canorus* [57] and *Falco amurensis* [58], with abundant rainfall from spring to early autumn in its latter part. The route taken by *pekinensis*, after leaving Africa, however, detours through the semi-arid regions of southwest, central and eastern Asia, thus flying through almost their entire distribution range. Along the way, they bypass the mountainous barrier from the Tienshan Mountains to the Himalayas through the Junggar Basin – an important corridor for birds using Central Asian and East African flyways, e.g. the threatened MacQueen's bustard (*Chlamydotis macqueenii*) [59]. This detour through suitable environments and tracing the subspecies’ range may support the hypothesis that the migration route might reflect the historical expansion of *pekinensis* eastward along this same route, which has been hypothesised but will need more evidence to be verified [60]. On this premise, they may have remained isolated from the nominate *apus* in an arid refugium during the glacial period before the eastward population expansion. In the case of the nominate *apus*, we speculate that its ancestral populations colonized Europe via the west coast of Africa, from a relatively wet refugium, bypassing their greatest barrier – the Sahara Desert. However, these scenarios must be inferred by coupling genomic-based historical demographic analysis and paleoclimate reconstruction[8]. Such an approach seizes a good opportunity to understand the roles of population expansion of *pekinensis* along this route and prior isolation from the nominate *apus* in an arid refugium during the evolution of the two subspecies.

At present, only a single population of *pekinensis* has been studied. We suggest that future work should be directed to investigate migration patterns in multiple populations of *pekinensis* throughout its geographical range in central and western Asia. While exploring these populations, parallel studies allow us to compare whether chain migration is also present in *pekinensis* [22],and to what extent nonbreeding grounds of other *pekinensis* populations would geographically overlap with the nominate *apus* or the Beijing *pekinensis* population studied here. Moreover, it would be extremely intriguing to delineate migration routes of populations at the contact zones (i.e. from Iran to central Kazakhstan), in order to determine whether a migratory divide can act as barrier to maintain population divergence between the nominate *apus* and *pekinensis* [5].

Using light level geolocation data not only sheds new lights on the migration of East Asian *pekinensis* of the Common Swift, also advances our knowledge of other migratory swift species in East Asia. Only recently, migration patterns of two East Asian breeding swifts,i.e. Pacific Swift *Apus pacificus* [16] and White-throated Needletail *Hirundapus caudacutus* [17] have been uncovered. It is not surprising that their migratory routes and wintering grounds follow the East Asian-Australasian Flyway given their non-breeding ranges in SE Asia and Australia. Unlike *pekinensis*, it is worth noting that their non-stop flight involves crossing the airspace over oceans. The formation of migratory patterns (e.g. the choice of migratory routes and wintering grounds) of Pacific Swift and White-throated Needletail may reflect an adaptation to explore forest habitats en route in a tropical climate [16, 17].

## Conclusions

In conclusion, our results painted a picture of the poor-known migration progress of *pekinensis* tracked from Beijing, which travelled the 14,000 km one-way route through eastern and central Asia to south-western Africa each year. Compared with the nominate *apus*, the tracked *pekinensis* experienced more arid environmental conditions and habitats during the non-breeding periods. This pattern might indicate that the subspecies *pekinensis* of the Common Swift has adapted to explore arid regions at certain stages of their annual cycle, and might be correlative with the historical colonisation route by tracking preferred habitats from sub-Saharan Africa into Asia. However, whether this specialization in habitat preference, together with other possible environmental variables, could be part of the explanation as to why *pekinensis* diverged from nominate *apus* in their evolutionary history remains to be tested. The two subspecies may thus prove to be good models for further comparative studies of the specialized intrinsic genetic, behavioural and physiological mechanisms that allow swifts to manage a highly mobile life-style spending substantial part of the year constantly on the wing [61].

## Supplementary Information

**Additional file 1. Table S1**. Distribution of data over years.** Table S2**. Key phenological parameters of migration of different sex individuals of *A. a. pekinensis* breeding in Beijing.** Table S3**. Results of comparative analysis of characteristic parameters between female and male *pekinensis* using t-test.** Table S4**. Results of comparative analysis of characteristic parameters between *pekinensis* from Beijing (N=25) and nominate *apus* from Sweden (N=25) using t-test.** Table S5**. Monthly and annual precipitation (mm) at several sites reported in the breeding areas of both subspecies.

**Additional file 2. Table S6**. Migration phenology of each individual in this study.

**Additional file 3. Figure S1**. The breeding and wintering areas of two subspecies of Common Swift and the random sample points used in this study.

**Additional file 4. Table S7**. List of random samples and monthly precipitation (mm) during the breeding season.

**Additional file 5. Table S8**. Monthly precipitation (mm) at several sites reported within the wintering areas of both subspecies.

**Additional file 6. Table S9**. List of random samples and monthly precipitation (mm) during the wintering season.

**Additional file 7. Figure S2**. Map showing the tracks away from breeding site and across the Junggar Basin.

**Additional file 8. Figure S3**. Maps showing the migration routes of five individuals in two years. The pentagram represents the bird breeding site and fieldwork location -Beijing. The dotted lines indicate the lack of data in the two weeks before/after the autumn/spring equinoxes. The yellow lines represent the autumn routes, the blue line represent the spring routes.

## Data Availability

The datasets supporting the study of this article were included within the additional files. The raw light data are available from the corresponding author.
