# Supplementary material for: A 30,000-km journey by Apus apus pekinensis tracks arid lands between northern China and south-western Africa
Source: Mov Ecol. 2022 Jun 29;10:29. doi: 10.1186/s40462-022-00329-2 (PMC9245314; doi:10.1186/s40462-022-00329-2)
Supplement: Supplementary file 1 — Additional file 1. Table S1. Distribution of data over years. Table S2. Key phenological parameters of migration of different sex individuals of A. a. pekinensis breeding in Beijing. Table S3. Results of comparative analysis of characteristic parameters between female and male pekinensis using t-test. Table S4. Results of comparative analysis of characteristic parameters between pekinensis from Beijing (N=25) and nominate apus from Sweden (N=25) using t-test. Table S5. Monthly and annual precipitation (mm) at several sites reported in the breeding areas of both subspecies. [file 40462_2022_329_MOESM1_ESM.docx]

Table S1. Distribution of data over years. Two individuals (one male and one female) first-tagged in 2014 were recaptured both in 2015 and 2016, one male first-tagged in 2015 was recaptured both in 2016 and 2017, and one individual of unknown sex first-tagged in 2016 was recaptured in 2017 and 2018. There were four individuals having unknown sex due to missing blood samples.

| Tag date | Recapture date | No. individuals | No. males | No. females | No. unknown sex |
| --- | --- | --- | --- | --- | --- |
| 2014.5.24 | 2015.5.24 | 13 | 10 | 3 | 0 |
| 2014.5.24 | 2016.5.21 | 3 | 1 | 2 | 0 |
| 2015.5.24 | 2016.5.21 | 7 | 4 | 2 | 1 |
| 2015.5.24 | 2017.5.21 | 1 | 1 | 0 | 0 |
| 2016.5.21 | 2017.5.20 | 2 | 0 | 0 | 2 |
| 2016.5.21 | 2018.5.19 | 3 | 1 | 0 | 2 |

Table S2. Key phenological parameters of migration of different sex individuals of *A. a. pekinensis* breeding in Beijing.

| **Females (N=6)** | Mean±SD | Range |
| --- | --- | --- |
| **Autumn migration** |  |  |
| Departure from Beijing | Jul 18^th^±5 | Jul 12^th^-Jul 25^th^ |
| Travel time (days) | 47±13 | 30-60 |
| No. stopover sites | 4±1 | 2-5 |
| Stopover time (days) | 69±24 | 39-104 |
| Total duration (days) | 114±18 | 89-134 |
| Migration distance (km) | 14579±1001 | 13432-15902 |
| Direct distance (km) | 11721±291 | 11404-12182 |
| Detour (%) | 24.41±8.22 | 15.40-35.56 |
| Travel speed (km/day) | 348±112 | 228-518 |
| Migration speed (km/day) | 128±18 | 102-153 |
| Arrival at wintering area | Nov 8^th^±15 | Oct 22^nd^-Nov 24^th^ |
| Duration of wintering period (days) | 91±16 | 76-114 |
| **Spring migration** |  |  |
| Departure from wintering area | Feb 7^th^±4 | Feb 1^st^-Feb 13^st^ |
| Travel time (days) | 27±9 | 18-45 |
| No. stopover sites | 1±1 | 1-2 |
| Stopover time (days) | 44±12 | 25-58 |
| Total duration (days) | 71±7 | 63-83 |
| Migration distance (km) | 14285±1007 | 13658-16321 |
| Direct distance (km) | 11792±221 | 11390-11984 |
| Detour (%) | 21.11±7.72 | 16.63-36.36 |
| Travel speed (km/day) | 567±154 | 307-771 |
| Migration speed (km/day) | 207±18 | 186-236 |
| Arrival at Beijing | Apr 19^th^±10 | Apr 9^th^-May 7^th^ |
| Duration in Beijing (days) | 90±8 | 79-100 |
| **Males (N=15)** |  |  |
| **Autumn migration** |  |  |
| Departure from Beijing | Jul 17^th^±7 | Jul 3^rd^-Jul 25^th^ |
| Travel time (days) | 39±14 | 25-69 |
| No. stopover sites | 4±1 | 2-6 |
| Stopover time (days) | 70±11 | 43-86 |
| Total duration (days) | 109±13 | 92-130 |
| Migration distance (km) | 14837±767 | 14052-16096 |
| Direct distance (km) | 11740±342 | 11289-12360 |
| Detour (%) | 26.42±6.49 | 15.35-37.31 |
| Travel speed (km/day) | 426±140 | 198-646 |
| Migration speed (km/day) | 138±18 | 113-170 |
| Arrival at wintering area | Nov 3^th^±10 | Oct 17^th^-Nov 17^th^ |
| Duration of wintering period (days) | 100±16 | 67-139 |
| **Spring migration** |  |  |
| Departure from wintering area | Feb 11^th^±11 | Jan 16^th^-Mar 5^th^ |
| Travel time (days) | 30±12 | 14-56 |
| No. stopover sites | 2±1 | 1-4 |
| Stopover time (days) | 36±9 | 26-53 |
| Total duration (days) | 65±10 | 51-90 |
| Migration distance (km) | 13552±917 | 12488-15512 |
| Direct distance (km) | 11825±312 | 11202-12328 |
| Detour (%) | 14.60±6.97 | 5.63-26.66 |
| Travel speed (km/day) | 522±187 | 229-910 |
| Migration speed (km/day) | 210±27 | 156-276 |
| Arrival at Beijing | Apr 17^th^±9 | Apr 7^th^-May 14^th^ |
| Duration in Beijing (days) | 90±10 | 71-109 |

Table S3. Results of comparative analysis of characteristic parameters between female (N=6) and male (N=15) Beijing Swifts using t-test. There was no sexual difference on the timing, distances, durations, or speeds of migration. As a sexually monomorphic species, Beijing swifts didn’t show significant sexual difference on the date of arrival at the breeding season.

|  | t | df | p-value |
| --- | --- | --- | --- |
| **Autumn migration** |  |  |  |
| Departure from Beijing | 0.32126 | 11.96 | 0.7536 |
| Travel time | 1.0195 | 10.185 | 0.3316 |
| No. stopover sites | -0.91652 | 11.571 | 0.3781 |
| Stopover time | -0.10001 | 5.9027 | 0.9236 |
| Total duration | 0.56054 | 7.024 | 0.5925 |
| Migration distance | -0.56774 | 7.4741 | 0.5869 |
| Detour | -0.53761 | 7.628 | 0.6062 |
| Travel speed | -1.3443 | 11.518 | 0.2047 |
| Migration speed | -0.89825 | 9.8499 | 0.3905 |
| Arrival at wintering area | 0.84698 | 6.9207 | 0.4253 |
| Duration of wintering period | -1.283 | 9.4179 | 0.2302 |
| **Spring migration** |  |  |  |
| Departure from wintering area | -1.3406 | 18.997 | 0.1959 |
| Travel time | -0.50854 | 12.285 | 0.6201 |
| No. stopover sites | -0.63145 | 16.283 | 0.5365 |
| Stopover time | 1.4762 | 7.4111 | 0.1811 |
| Total duration | 1.5313 | 13.995 | 0.148 |
| Migration distance | 1.5451 | 8.5311 | 0.1586 |
| Detour | 1.7956 | 8.4639 | 0.1082 |
| Travel speed | 0.56496 | 11.206 | 0.5832 |
| Migration speed | -0.34068 | 13.65 | 0.7385 |
| Arrival at Beijing | 0.25448 | 8.5316 | 0.8052 |
| Duration in Beijing | -0.061479 | 11.739 | 0.952 |

Table S4. Results of comparative analysis of characteristic parameters between *pekinensis* from Beijing (N=25) and nominate *apus* from Sweden (N=25) using t-test.

|  | t | df | p-value |
| --- | --- | --- | --- |
| **Autumn migration** |  |  |  |
| Travel time | 5.3568 | 40.748 | **3.60E-06** |
| Stopover time | 8.9434 | 44.95 | **1.54E-11** |
| Stopover rate | 3.503 | 38.268 | **0.001188** |
| Total duration | 12.28 | 40.412 | **3.21e-15** |
| Migration distance | 18.068 | 41.19 | **<2.20e-16** |
| Travel speed | -1.0124 | 45.684 | 0.3167 |
| Migration speed | -5.1161 | 25.587 | **2.59e-05** |
| Duration of wintering period | -20.306 | 43.476 | **<2.20e-16** |
| **Spring migration** |  |  |  |
| Travel time | 6.637 | 35.779 | **1.02e-07** |
| Stopover time | 11.402 | 30.06 | **1.93e-12** |
| Stopover rate | 5.6255 | 47.991 | **9.32e-07** |
| Total duration | 16.857 | 42.912 | **<2.20e-16** |
| Migration distance | 20.48 | 46.16 | **<2.20e-16** |
| Travel speed | -3.8709 | 46.695 | **3.36e-04** |
| Migration speed | -8.7202 | 26.033 | **3.35e-09** |
| Duration of breeding period | 5.7896 | 47.53 | **5.41e-07** |

Table S5 Monthly and annual precipitation (mm) at several sites reported in the breeding areas of both subspecies.

| Subspecies | Country | Site | Longitude | Latitude | Annual | Breeding Period | April | May | June | July | August |
| --- | --- | --- | --- | --- | --- | --- | --- | --- | --- | --- | --- |
| apus (north) | Finland | Lammi | 25.55 | 61.17 | 606.04 | Jun-Aug | -- | -- | 60.61 | 64.83 | 89.25 |
| apus (north) | Finland | Harju | 27.55 | 60.55 | 726.41 | Jun-Aug | -- | -- | 55.89 | 62.14 | 94.08 |
| apus (north) | Sweden | Hakkas | 21.55 | 66.92 | 531.79 | Jun-Aug | -- | -- | 54.43 | 68.25 | 81.54 |
| apus (north) | Sweden | Falun | 15.78 | 60.55 | 589.30 | Jun-Aug | -- | -- | 55.38 | 59.31 | 76.68 |
| apus (north) | Sweden | Barkö | 18.26 | 60.28 | 591.52 | Jun-Aug | -- | -- | 55.29 | 54.38 | 74.77 |
| apus (north) | Sweden | Ås | 16.45 | 56.24 | 497.83 | Jun-Aug | -- | -- | 38.96 | 42.67 | 52.52 |
| apus (north) | Sweden | Lund | 13.21 | 55.71 | 736.15 | Jun-Aug | -- | -- | 53.85 | 68.52 | 73.43 |
| apus (north) | Sweden | Skurup | 13.5 | 55.47 | 749.44 | Jun-Aug | -- | -- | 56.07 | 71.90 | 66.32 |
| apus (south) | UK | Great Yarmouth | 1.66 | 52.59 | 624.55 | May-Jul | -- | 48.00 | 41.36 | 51.62 | -- |
| apus (south) | UK | Fowlmere | 0.06 | 52.08 | 640.51 | May-Jul | -- | 57.02 | 42.97 | 48.29 | -- |
| apus (south) | Netherlands | Groesbeek | 5.94 | 51.78 | 778.21 | May-Jul | -- | 56.86 | 59.82 | 76.49 | -- |
| apus (south) | Belgium | Gent | 3.73 | 51.08 | 834.06 | May-Jul | -- | 63.12 | 63.50 | 79.45 | -- |
| apus (south) | Belgium | Hechtel | 3.81 | 51.2 | 821.27 | May-Jul | -- | 62.69 | 65.54 | 78.22 | -- |
| apus (south) | Belgium | Melsele | 4.28 | 51.22 | 816.93 | May-Jul | -- | 61.64 | 62.86 | 78.73 | -- |
| apus (south) | Germany | Kronberg | 8.52 | 50.18 | 690.72 | May-Jul | -- | 62.73 | 60.89 | 71.81 | -- |
| apus (south) | Czech | Pečky | 15.03 | 50.09 | 493.94 | May-Jul | -- | 60.86 | 56.47 | 77.69 | -- |
| apus (south) | Italy | Modena | 10.95 | 44.39 | 701.78 | May-Jul | -- | 53.66 | 60.81 | 44.61 | -- |
| apus (south) | Spain | Guipúzcoa | -1.79 | 43.34 | 1305.11 | May-Jul | -- | 69.79 | 75.63 | 74.74 | -- |
| apus (south) | Spain | Lugo | -7.56 | 43.01 | 1428.71 | May-Jul | -- | 83.80 | 64.50 | 29.89 | -- |
| apus (south) | Spain | León | -5.61 | 42.66 | 581.06 | May-Jul | -- | 44.09 | 42.82 | 25.82 | -- |
| apus (south) | Spain | Barcelona | 2.26 | 41.93 | 718.53 | May-Jul | -- | 66.28 | 51.98 | 59.63 | -- |
| apus (south) | Spain | Segovia | -4.11 | 40.95 | 462.57 | May-Jul | -- | 39.68 | 31.12 | 19.33 | -- |
| apus (south) | Spain | Madrid | -3.24 | 40.37 | 416.46 | May-Jul | -- | 28.36 | 20.75 | 11.37 | -- |
| apus (south) | Spain | Ciudad Real | -3.93 | 38.99 | 360.51 | May-Jul | -- | 27.99 | 14.63 | 6.24 | -- |
| pekinensis | China | Beijing | 116.27 | 39.99 | 559.98 | Apr-Jul | 17.51 | 26.94 | 79.49 | 204.19 | -- |
| pekinensis | China | Datong | 113.17 | 40.06 | 357.89 | Apr-Jul | 20.22 | 30.01 | 44.38 | 104.42 | -- |
| pekinensis | China | Qingzhou | 118.28 | 36.42 | 776.40 | Apr-Jul | 52.60 | 46.35 | 99.19 | 209.72 | -- |
| pekinensis | China | Huhhot | 111.41 | 40.48 | 322.53 | Apr-Jul | 18.62 | 27.55 | 38.43 | 89.81 | -- |
