# Supplementary figures and images for: A 30,000-km journey by Apus apus pekinensis tracks arid lands between northern China and south-western Africa
Source: Mov Ecol. 2022 Jun 29;10:29. doi: 10.1186/s40462-022-00329-2 (PMC9245314; doi:10.1186/s40462-022-00329-2)

Figure S1

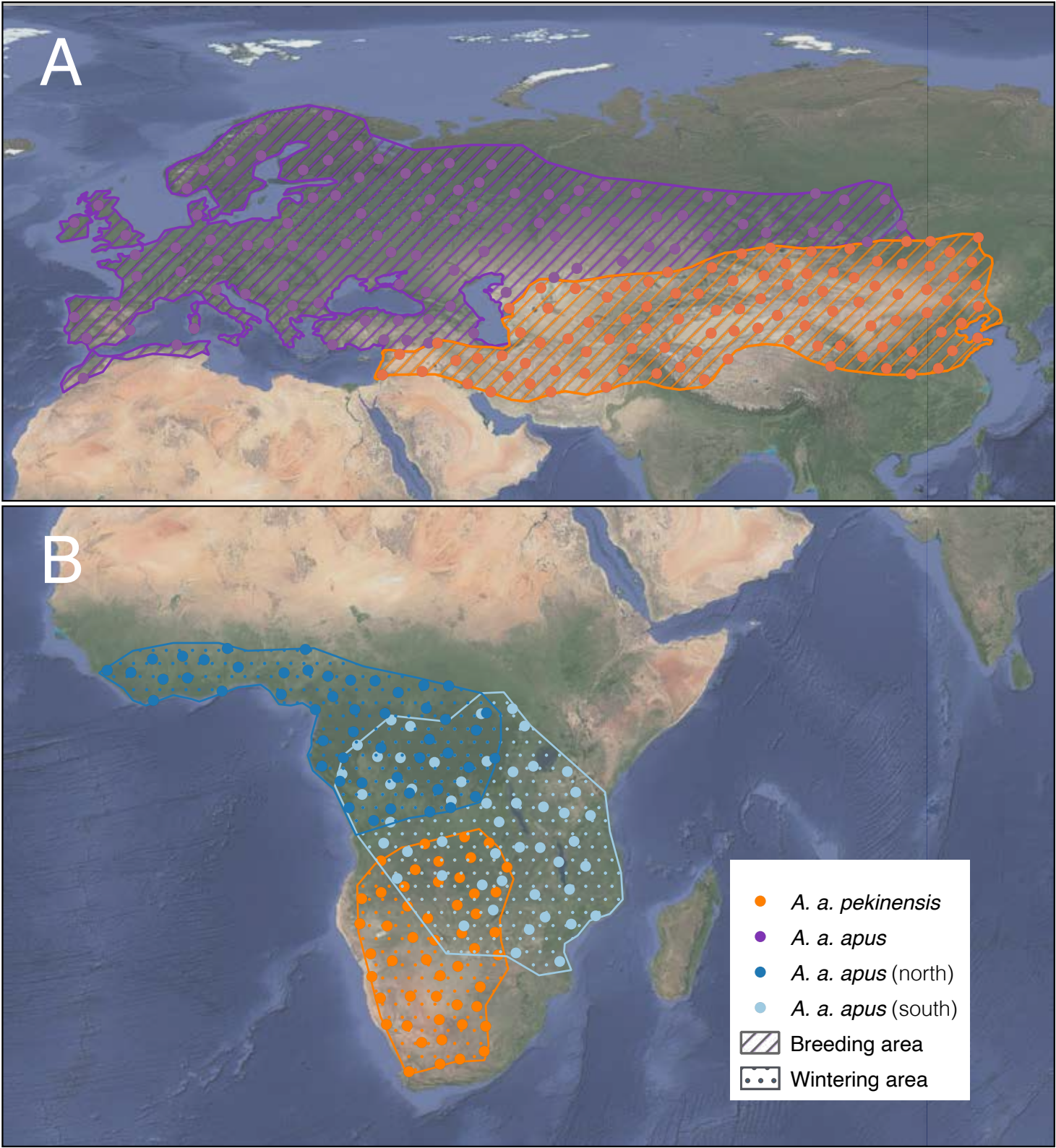

Supplement: Supplementary file 3 — Additional file 3. Figure S1. The breeding and wintering areas of two subspecies of Common Swift and the random sample points used in this study. [file 40462_2022_329_MOESM3_ESM.pdf]

Figure S2

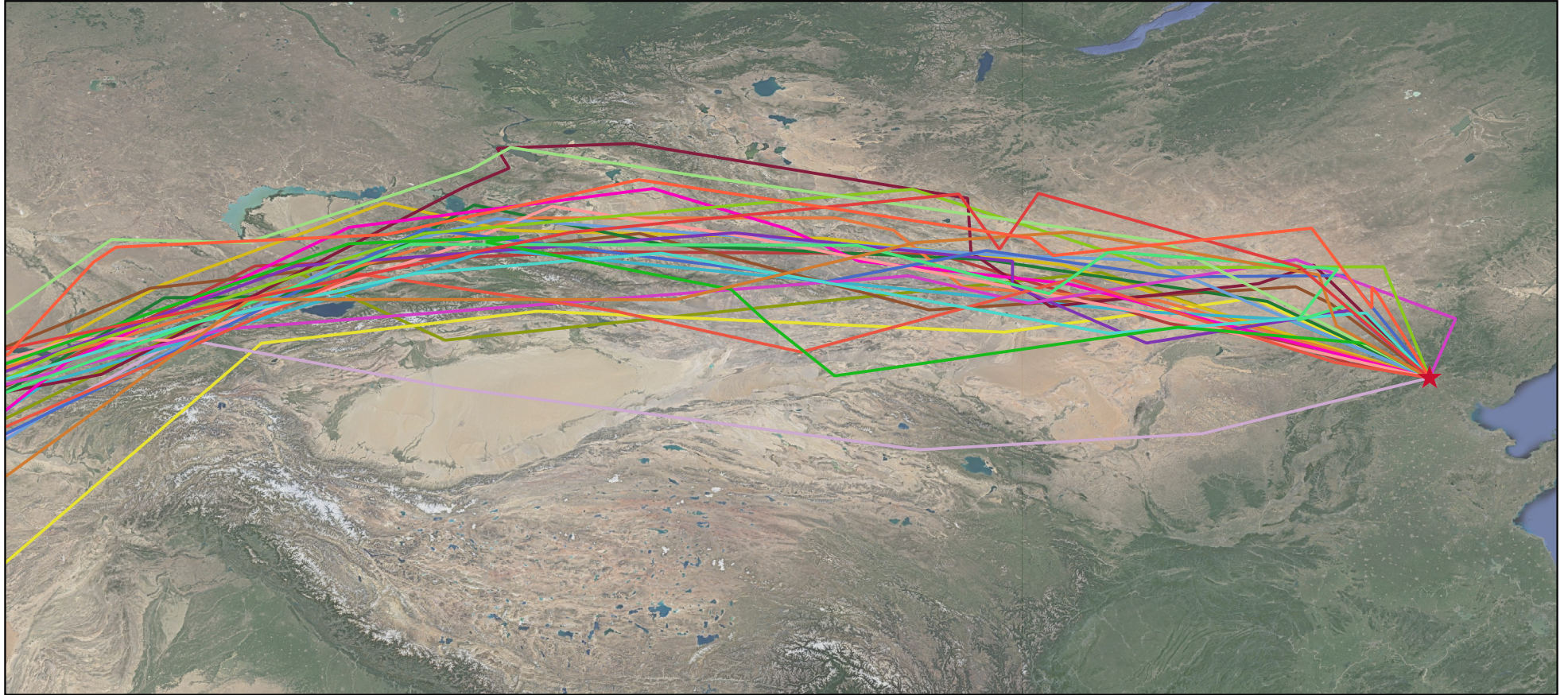

Supplement: Supplementary file 7 — Additional file 7. Figure S2. Map showing the tracks away from breeding site and across the Junggar Basin. [file 40462_2022_329_MOESM7_ESM.pdf]

Figure S3

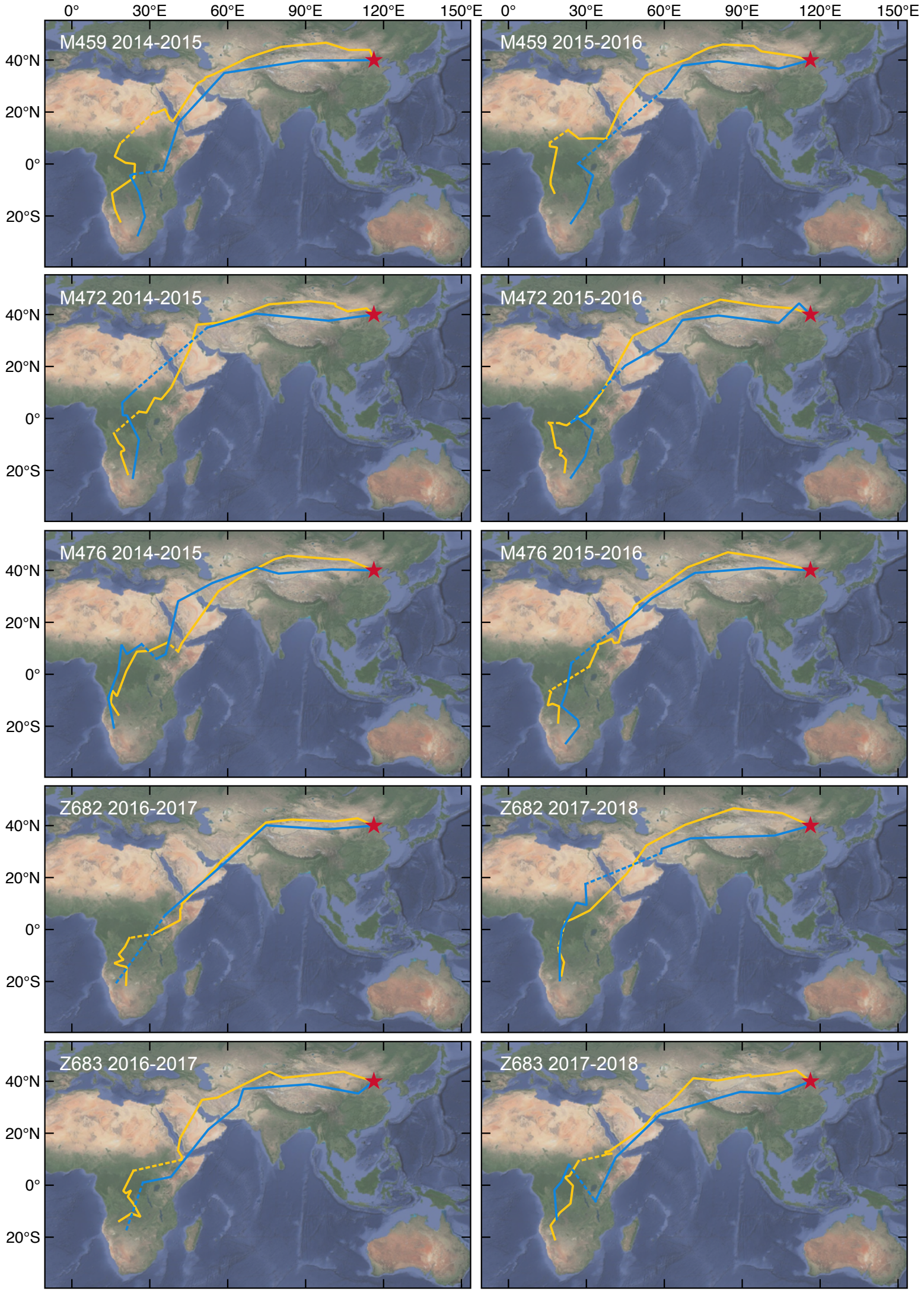

Supplement: Supplementary file 8 — Additional file 8. Figure S3. Maps showing the migration routes of five individuals in two years. The pentagram represents the bird breeding site and fieldwork location -Beijing. The dotted lines indicate the lack of data in the two weeks before/after the autumn/spring equinoxes. The yellow lines represent the autumn routes, the blue line represent the spring routes. [file 40462_2022_329_MOESM8_ESM.pdf]
